# Effectiveness of CO_2_ laser therapy in treating acne depressed scar: A protocol of systematic review

**DOI:** 10.1097/MD.0000000000023732

**Published:** 2021-01-22

**Authors:** Huan Zang, Ya-nan Xu

**Affiliations:** a Department of Dermatology, Urumqi Maternal and Child Health Hospital, Urumqi, Xinjiang; b Department of Dermatology, Yan’an People's Hospital, Yan’an, Shaanxi, China.

**Keywords:** acne depressed scar, CO_2_ laser therapy, effectiveness, randomized controlled trial, safety

## Abstract

**Background:**

This study is to assess the effectiveness of CO_2_ laser therapy (COLT) in treating patients with acne depressed scar (ADS).

**Methods:**

Relevant randomized controlled trials will be checked by search the electronic databases of Cochrane Library, PUBEMD, EMBASE, Web of Science, Allied and Complementary Medicine Database, VIP Database, CBM database, and China National Knowledge Infrastructure. All potential randomized controlled trials of COLT for patients with ADS will be identified by 2 independent authors by searching all sources from inception to present. Two authors will independently undertake literature selection, data collection and study quality assessment. Any divergences between 2 authors will be settled down by a third author through discussion. RevMan 5.3 software will be used for statistical analysis.

**Results:**

This study will assess the effectiveness of COLT for patients with ADS.

**Conclusions:**

This study may provide helpful evidence to determine whether COLT is an effective intervention for patients with ADS.

**Study registration:**

OSF (osf.io/m9ghv).

## 1 Introduction

Acne depressed scar (ADS) is a common type of facial skin disorder.^[1–6]^ It is estimated that the prevalence of postacne scar varies from 11% to 14% in the general population, and 49% of them affected with acne.^[7,8]^ Although it is not a lethal disease for patients with ADS, they often suffer from negative psychosocial problems, which significantly affect their quality of life.^[9,10]^ Fortunately, CO_2_ laser therapy (COLT) is reported to treat ADS effectively.^[11–25]^ However, no systematic review has been carried out assess the effectiveness of COLT for patients with ADS. Thus, this study will point out the existing gap and will systematically summarize the clinical evidence of COLT in treating ADS.

## 2 Methods

### 2.1 Study registration

This protocol has been funded and registered through OSF (osf.io/m9ghv). We will report it according to the Preferred Reporting Items for Systematic Review and Meta-Analysis Protocols statement.^[26]^

### 2.2 Criteria for including studies

#### 2.2.1 Types of studies

All randomized controlled trials of COLT for patients with ADS will be included with no limitations to language and publication time.

#### 2.2.2 Types of interventions

In the experimental group, we will include all involved studies that focus on COLT management.

In the control group, control interventions could be any treatment, except any forms of COLT.

#### 2.2.3 Types of participants

We will only include subjects who had a confirmed clinical diagnosis of ADS. No limitations upon the race, gender, age, economic status, and educational background will be placed.

#### 2.2.4 Types of outcome measurements

The primary outcome is severity of acne scars, as measured by Echelle D’Evaluation Clinique des Cicatrices D’Acne grading scale or other associated scales.

The secondary outcomes are scar improvement (as assessed by Vancouver Scar Scale or other scales), crust time, time to complete molting, edema time, erythema duration, depression (as checked by Self-rating Depression Scale or other scores), anxiety (as evaluated by Self-rating Anxiety Scale or other tools), quality of life (as identified by Global Quality of Life Scale or other scales), and adverse events.

### 2.3 Search strategy

#### 2.3.1 Electronic databases sources

We will search all following electronic databases from the beginning of each 1 to the present: Cochrane Library, PUBEMD, EMBASE, Web of Science, Allied and Complementary Medicine Database, VIP Database, CBM database, and China National Knowledge Infrastructure. All electronic databases will be searched without restrictions to language and publication time by 2 independent authors. If any different views occur, a third author will help to solve them through discussion. The detailed strategy for searching the Cochrane Library is shown in Table 1. We will also build detailed search strategies for any other electronic databases.

**Table 1:** Search strategy sample of Cochrane Library.

Number	Search terms
1	MeSH descriptor: (acne vulgaris) explode all trees
2	((acne^∗^) or (depressed scar^∗^) or (scar^∗^) or (acne scar^∗^) or (atrophic scar^∗^) or (sunken scar^∗^) or (facial scar^∗^)):ti, ab, kw
3	Or 1-2
4	MeSH descriptor: (lasers, gas) explode all trees
5	(CO2 laser therapy) explode all trees
6	((CO2 laser^∗^) or (carbon dioxide laser^∗^) or (treatment^∗^) or (therapy^∗^) or (intervention^∗^)):ti, ab, kw
7	Or 4-6
8	MeSH descriptor: (randomized controlled trials) explode all trees
9	MeSH descriptor: (clinical trials as topic) explode all trees
10	((random^∗^) or (randomly^∗^) or (blind^∗^) or (allocation^∗^) or (placebo^∗^) or (sham^∗^) or (control^∗^) or (comparator^∗^)):ti, ab, kw
11	Or 8-10
12	3 and 7 and 11

#### 2.3.2 Other literature sources

We will also search conference papers, clinical trial registries, and reference lists of relevant reviews.

### 2.4 Study selection

All retrieved literatures will be imported to EndNote 7.0, and we will exclude any duplicate records. Two authors will independently scan the titles and abstracts of all searched studies. Then, they will read full-texts of any unclear studies to determine whether they fit the final inclusion criteria. If there are disagreements between 2 authors, a third independent author will work as an arbitrator and ultimately make the decision. The study selection procedure will be shown in the flow diagram.

### 2.5 Data extraction and management

Two authors will extract data from all selected studies independently. Any discrepancies between 2 authors will be solved through discussion by a third author. This data collect form consists of reference identification, first author, year of publication, basic characteristics of patient, diagnostic criteria, inclusion and exclusion criteria, randomization, blinding, intervention and control indicators, outcome measurements, research results, follow-up information, adverse events, and other detailed information. If necessary, we will contact primary trial author for further information when we find insufficient or missing data.

### 2.6 Risk of bias assessment

Two independent authors will identify the risk of bias for each eligible trial using Cochrane risk of bias tool. It covers 7 aspects and each 1 will be classified as low risk of bias, unclear risk of bias, or high risk of bias. Any disagreements between 2 authors will be resolved by a third author through arbitration.

### 2.7 Statistical analysis

#### 2.7.1 Data synthesis

RevMan 5.3 software will be used for statistical analysis. We will express enumeration data with risk ratio and 95% confidence intervals, and measurement data with mean difference or standardized mean difference and 95% confidence intervals. We will use *I*^*2*^ statistic test to determine the heterogeneity of the research results. If *I*^*2*^ ≤50%, the heterogeneity among the studies will be considered as acceptable; and a fixed-effect model will be used. Otherwise, if *I*^*2*^ > 50%, it will be considered as having substantial heterogeneity among the trials, and a random-effect model will be utilized. We will undertake meta-analysis if there is not significant statistical heterogeneity in the outcome results. If we identify substantial heterogeneity, we will further explore the sources of obvious heterogeneity using subgroup analysis. In addition, we will present outcome results as descriptive summary.

#### 2.7.2 Subgroup analysis

If there is obvious heterogeneity among included trials, we will undertake a subgroup analysis in accordance with the basic information of study and patient, different managements, comparators, and outcomes.

#### 2.7.3 Sensitivity analysis

If possible, we will perform sensitivity analysis to verify the robustness of conclusions by removing low quality studies.

#### 2.7.4 Reporting bias

When necessary, we will investigate reporting bias using funnel plot and Egger regression test if more than 10 eligible studies are include.^[27,28]^

### 2.8 Grading the quality of evidence

We will evaluate the quality of evidence using Grading of Recommendations Assessment, Development and Evaluation. The quality will be graded as very low, low, moderate, or high level.^[29]^

### 2.9 Ethics and dissemination

This study does not inquire ethical approval, because we will not obtain individual patient data. This study is expected to be published through a peer-reviewed journal.

## 3 Discussion

In recent years, an increased number of clinical studies have explored the effectiveness and safety of COLT for patients with ADS.^[11–25]^ However, there is no systematic review to collect and evaluate that research evidence. In order to provide strong evidence-based medicine for clinical practice and further research of COLT in the treatment of ADS, we will perform this systematic review. This study will comprehensively collect evidence without restrictions, and methodological quality of all included evidence will be appraised by Cochrane risk of bias tool. The results of this study may provide precise empirical evidence of COLT for patients with ADS for clinical practice.

## Author contributions

**Conceptualization:** Huan Zang, Ya-nan Xu.

**Data curation:** Huan Zang, Ya-nan Xu.

**Formal analysis:** Huan Zang, Ya-nan Xu.

**Funding acquisition:** Ya-nan Xu.

**Investigation:** Ya-nan Xu.

**Methodology:** Huan Zang.

**Project administration:** Ya-nan Xu.

**Resources:** Huan Zang, Ya-nan Xu.

**Software:** Huan Zang.

**Supervision:** Ya-nan Xu.

**Validation:** Huan Zang, Ya-nan Xu.

**Visualization:** Huan Zang, Ya-nan Xu.

**Writing – original draft:** Huan Zang, Ya-nan Xu.

**Writing – review & editing:** Huan Zang, Ya-nan Xu.
